# ALT Trends through Childhood and Adolescence Associated with Hepatic Steatosis at 24 Years: A Population-Based UK Cohort Study

**DOI:** 10.3390/children7090117

**Published:** 2020-09-01

**Authors:** Ahlia Sekkarie, Jean A. Welsh, Kate Northstone, Catherine E. Cioffi, Aryeh D. Stein, Janet Figueroa, Usha Ramakrishnan, Miriam B. Vos

**Affiliations:** 1 Laney Graduate School, Emory University, Atlanta, GA 30322, USA; jwelsh1@emory.edu (J.A.W.); aryeh.stein@emory.edu (A.D.S.); mvos@emory.edu (M.B.V.); 2 Department of Pediatrics, Emory School of Medicine, Atlanta, GA 30322, USA; catherine.cioffi@emory.edu (C.E.C.); janet.figueroa@emory.edu (J.F.); 3 Population Health Science, Bristol Medical School, Bristol BS8 2BN, UK; Kate.Northstone@bristol.ac.uk; 4 Hubert Department of Global Health, Rollins School of Public Health, Emory University, Atlanta, GA 30322, USA; uramakr@emory.edu

**Keywords:** NAFLD, ALT, childhood, longitudinal, ALSPAC

## Abstract

(1) Background: Alanine aminotransferase (ALT) is used to screen for non-alcoholic fatty liver disease (NAFLD) in children; however, the optimal age to commence screening is not determined. Our objective was to describe whether ALT trends from 9–24 years were associated with hepatic steatosis at 24 years in a population-based UK cohort. (2) Methods: The sample included 1156 participants who were assessed for hepatic steatosis at 24 years and had at least two ALT measurements at 9, 15, 17, and/or 24 years. Controlled attenuation parameter scores were used to assess steatosis (low (<248 dB/m), mild/moderate (248–279 dB/m), severe (>279 dB/m)). Sex-stratified mixed-effects models were constructed to assess the liver enzyme trends by steatosis level. (3) Results: The final sample was 41.4% male and 10.4% had severe steatosis. In both sexes, ALT trends from 9 to 24 years differed in those with low vs. severe steatosis at 24 years (*p* < 0.001). There was no evidence of differences prior to puberty. At 17 years, the low vs. severe geometric mean ratio (GMR) was 0.69, 95% CI: 0.57–0.85 in males and (0.81, 0.65–1.01) females. At 24 years, the GMR was (0.53, 0.42–0.66) in males and (0.67, 0.54–0.84) females. (4) Conclusions: Higher ALT concentration in adolescence was associated with hepatic steatosis at 24 years. The increased screening of adolescents could strengthen NAFLD prevention and treatment efforts.

## 1. Introduction

Non-alcoholic fatty liver disease (NAFLD) is the hepatic manifestation of metabolic syndrome [1]. NAFLD is defined as having steatosis involving greater than 5% hepatocytes, typically assessed by liver biopsy or imaging, in the absence of other causes of hepatic steatosis including heavy alcohol intake [2]. Established risk factors for NAFLD include age, male sex, and obesity. The prevalence of NAFLD has increased considerably over recent decades in youth in parallel with the rise of obesity [3]. This is concerning because pediatric NAFLD can progress to nonalcoholic steatohepatitis (NASH), which is characterized by inflammation, as well as cirrhosis and end stage liver disease in adulthood [4]. NAFLD is also associated with an increased risk of diabetes, cardiovascular disease and pregnancy complications [5,6].

The onset and subsequent natural history of NAFLD in childhood and adolescence is not well characterized [2]. Despite reports of infants with hepatic steatosis [7,8,9,10], little is known about the disease in pre-pubertal children. Most children with NAFLD typically present clinically between 10 to 13 years old [11]. If the disease is caught early, outcomes can be improved through drugs or lifestyle changes, primarily by improving diet quality (e.g., reducing sugar) and increasing physical activity [2,12]. Therefore, the ability to detect NAFLD in its earliest stages is crucial to mitigating the consequences of childhood NAFLD.

Currently, the recommended screening method for NAFLD in children includes elevated alanine aminotransferase (ALT) serum concentration, concurrent with obesity and other risk factors for metabolic disease, such as a family history of diabetes [2]. While the other two primary liver enzymes, aspartate aminotransferase (AST) and gamma-glutamyl transferase (GGT) are not used as screening markers for NAFLD in children, they are associated with a worse histology of the disease when they are elevated along with ALT [2,13].

Due to the sparsity of studies on the incidence and natural history of pediatric NAFLD, especially prior to its diagnosis, the ideal age for screening for NAFLD is not known [2]. No studies using serial measurements have been published on the trajectory of liver enzymes from childhood to adulthood. Additionally, the association between liver enzyme trends from childhood into adulthood and the risk of NAFLD has not been previously explored. In this paper, we describe the trends in ALT and other liver enzyme concentrations in the UK population-based Avon Longitudinal Study of Parents and Children (ALSPAC) birth cohort from 9 to 24 years and determine whether these trends differ by category of hepatic steatosis at 24 years.

## 2. Materials and Methods

### 2.1. Study Design and Population

We used the data from a population-based birth cohort study (ALSPAC) based at the University of Bristol (Bristol, UK) that has previously been described in detail [14,15,16]. Briefly, ALSPAC enrolled 15,454 pregnant women in the greater Bristol area with expected delivery dates between 1 April 1991 and 31 December 1992. Of their children, 14,901 were alive at one year of age. When the offspring were 24 years of age, 10,018 participants were invited to a clinic visit (that wave was known locally as Focus@24) between 5 June 2015 and 31 October 2017, which included the collection of biological samples and anthropometric measures, and 4021 attended. Other waves of fieldwork occurred when the participants were ages seven through 17 years. Data from the 24 year clinic were collected and managed using REDCap electronic data capture tools hosted at the University of Bristol [17,18]. The study website contains details of all the data that are available through a fully searchable data dictionary and variable search tool [19].

Ethical approval for the study was obtained from the ALSPAC Ethics and Law Committee and The data from the 24 Year Clinic was approved by the National Research Ethics Service Committee South West—Frenchay: 14/SW/1173 ALSPAC Focus at 24+ (24 February 2015, confirmed 20 March 2015) Informed consent for the use of collected data via questionnaires and clinics was obtained from participants following the recommendations of the ALSPAC Ethics and Law Committee at the time.

### 2.2. Assessment of Liver Outcomes

Participants were asked to fast overnight or for at least six hours prior to phlebotomy and transient elastography. Blood samples were immediately centrifuged and frozen at −80 °C. Fasting serum ALT, AST, and GGT liver enzyme concentrations were obtained through standard clinical chemistry assays at ages 9 (non-fasting), 15, 17 and 24 years as previously described [20]. At 24 years, elevated ALT was defined as >19 U/L in women and >30 U/L in men [21].

At 24 years old, the participants were assessed by transient elastography for the non-invasive quantification of liver steatosis and fibrosis (FibroScan 502 Touch, Echosens, Paris, France). Individuals with an active implant, liver ascites, or who were pregnant were excluded from the liver scan. Transient elastography provides a controlled attenuation parameter (CAP) measure of steatosis and a measure of liver stiffness to quantify fibrosis. Manufacturer and machine indications were used to determine whether the M or XL probe would be used to conduct the scan. Ten readings were required for each patient to derive a CAP score and fibrosis result. CAP values outside the 100–400 dB/m range were considered invalid and coded as missing. Additionally, median fibrosis results greater than or equal to 15 kPa or with an IQR to median ratio greater than or equal to 30% were considered invalid and coded as missing.

We categorized participants by level of steatosis at 24 years based on CAP scores and using cut-off values derived from a meta-analysis by Karlas, et al. [22]. Low steatosis (<10%) was defined as <248 dB/m, mild/moderate steatosis (10–66%) was defined as 248–279 dB/m, and severe steatosis (>66%) was defined as >279 dB/m. We categorized the fibrosis values into two groups. The first group included those with no fibrosis or portal fibrosis without septa (F0–F1, <7.9 kPA) and the second group included those with any fibrosis: portal fibrosis, septa, or cirrhosis (F2–F4, >7.9 kPA) [23].

### 2.3. Covariates

We calculated the body mass index (BMI) as weight in kilograms divided by height in meters squared [24]. We classified BMI at 24 years as underweight (<18.5 kg/m^2^), normal weight (18.5 to <25 kg/m^2^), overweight (25 to <30 kg/m^2^), and obese (≥30 kg/m^2^). We used the highest level of maternal education reported during pregnancy as a proxy for socioeconomic status [25]. Mothers self-reported one of five categories: none/CSE (certificate of secondary education), vocational (vocational courses after 16 years of age), O (ordinary level exams at 16 years), A (advanced level exams at 18 years), and university degree and above [26]. Ethnicity was based on maternal ethnicity and categorized as White or Other.

### 2.4. Inclusion/Exclusion

We included all participants that had liver enzyme measures (ALT, AST, and GGT serum concentrations) available at two of the four available time points (9, 15, 17, and 24 years) and that had valid transient elastography measures at 24 years. We excluded women who self-reported pregnancy at 17 or 24 years. We excluded the respondents with hazardous alcohol consumption defined by an Alcohol Use Disorder Identification Test for Consumption (AUDIT-C) score greater than or equal to 4 (women) and 5 (men) [27,28].

### 2.5. Statistical Analysis

We calculated the median and interquartile range (IQR) values for continuous variables and counts and percentages for categorical variables for the full sample and stratified by level of hepatic steatosis (low, mild/moderate, or severe) at 24 years. Kruskal–Wallis tests were used to compare differences in continuous variables across steatosis levels. Chi-squared tests were used to compare differences between categorical variables. For cell counts less than five, Fisher’s exact tests were used. We reported sex-stratified ALT, AST, and GGT medians and inter-quartile ranges by age and hepatic steatosis level at 24 years.

Due to non-normal distributions assessed by the Shapiro–Wilk test for normality, we analyzed ALT, AST, and GGT as log-transformed outcomes in all regression analyses. We used repeated-measures linear mixed models to assess the trend differences of log-transformed ALT, AST, and GGT levels from ages 9 to 24 years by hepatic steatosis level at 24 years. We also modeled differences of log-transformed ALT by fibrosis. We included the fixed effects for categorical age, random effects for the intercept, and an unstructured error covariance structure. To back-transform the log-transformed means from the models, we exponentiated them to geometric means. We then calculated geometric mean ratios (GMR) to assess the differences between steatosis levels at each age. Comparisons were made using Tukey-adjusted pairwise tests. We checked all model residuals for normality.

We decided to conduct a sex-stratified analysis a priori. Covariates were adjusted for in a stepwise manner, whereby model 1 was unadjusted, model 2 included BMI at age 24 years as a covariate, and model 3 included maternal education and ethnicity as covariates.

A sensitivity analysis was performed to compare the characteristics of the original cohort to those in our analytic sample. In addition, to understand the impact of alcohol consumption, we (1) analyzed the association between high AUDIT-C score and hepatic steatosis, and (2) ran the models with the AUDIT-C score as a covariate instead of an exclusion criterion.

We conducted statistical analyses in SAS version 9.4 (Cary, NC, USA).

## 3. Results

**Figure 1:**
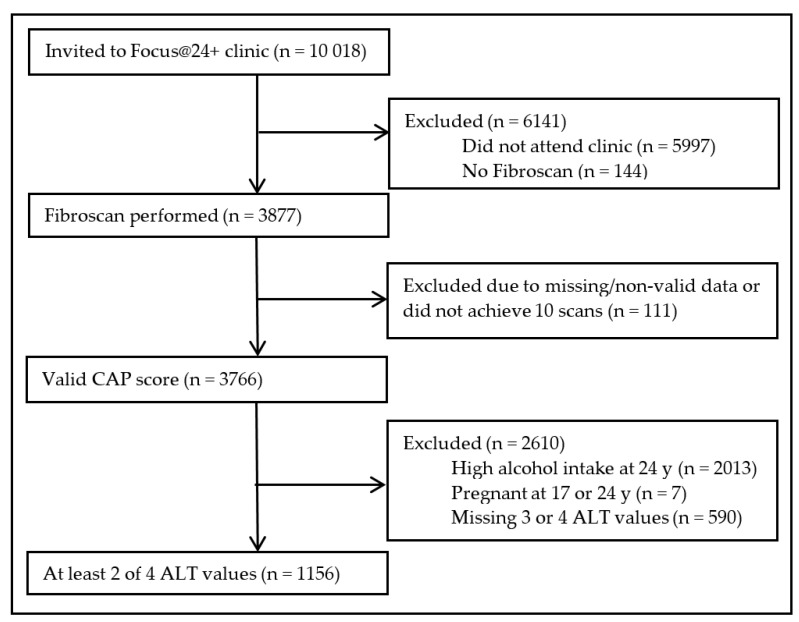
Flowchart of the participants included in final analysis. Of the 10,108 participants invited to Focus@24 year clinic, we excluded those that did not attend the clinic or did not get a liver scan because they were ineligible or excluded due to an active implant, liver ascites, or pregnancy. We also excluded participants with missing or non-valid controlled attenuation parameter (CAP) scores, who had a high alcohol intake, were pregnant at the 17 or 24 year clinic, and had less than 2 alanine aminotransferase (ALT) values measured at 9, 15, 17, and/or 24 years. Our final sample size was 1156.

Of the 10,018 active ALSPAC participants who were invited to participate in the Focus@24 + clinic, 3877 participants had Fibroscan performed (Figure 1). Of these, 3766 participants had a valid CAP score and 3600 participants had a valid fibrosis score. After exclusions for having ALT measures obtained on no or only one occasion (*n* = 590), pregnancy (*n* = 7), and high AUDIT-C score (*n* = 2013), our analytic sample size was 1156.

Selected demographic and clinical characteristics of the study sample are presented in Table 1. Most of the participants were female (58.6%) and reported being of White ethnicity (97.4%). The majority (77.8%) had low hepatic steatosis, but 11.9% had mild to moderate and 10.4% had severe hepatic steatosis (Table 1). There was a positive association between liver enzymes and hepatic steatosis at 24 years (*p* < 0.001). All the clinical biomarkers measured at 24 years had a positive association with hepatic steatosis, except for high-density lipoprotein (HDL) which had a negative association (*p* < 0.001). In sensitivity analysis, there was no association between high alcohol intake and hepatic steatosis level (Chi-square = 2.4, *p* = 0.30). Compared to those enrolled in the original cohort, participants in our sample were more likely to be female and have mothers with a higher education status (both *p* < 0.001).

**Figure 2:**
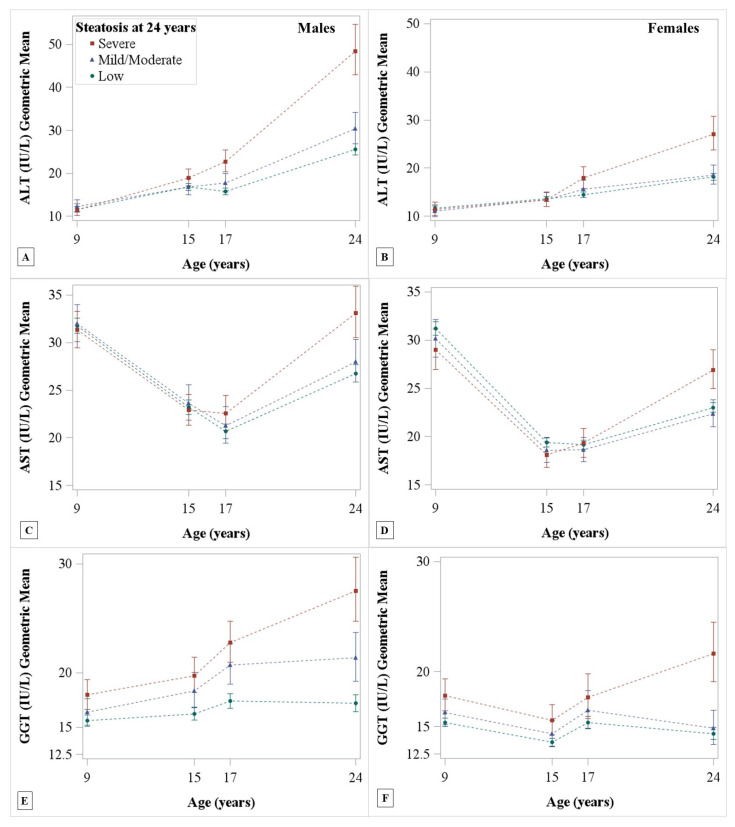
ALT, AST, GGT geometric mean and 95% CI trends by hepatic steatosis level and sex. Sample size was 479 males (**A**,**C**,**E**) and 677 females (**B**,**D**,**F**). Steatosis is defined from controlled attenuation parameter scores: low (<248 dB/m), mild/moderate (248–279 dB/m), severe (>279 dB/m). Low is marked by filled green circles, mild/moderate by blue filled triangles, and severe by filled red squares.

**Figure 3:**
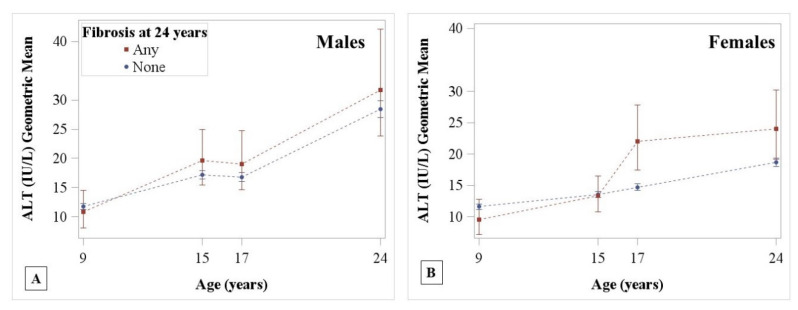
ALT geometric mean trends by fibrosis category and sex. (**A**) In males, and (**B**) in females. Any fibrosis (red square) includes those with portal fibrosis, septa, or cirrhosis (F2–F4, >7.9 kPA).

The trends of ALT, AST, and GGT over time differed across levels of steatosis for both sexes (Table 2). Figure 2 shows the geometric means and 95% CIs of each liver enzyme plotted over time and stratified by steatosis level at 24 years for each sex from model 1. ALT values increased with age in both sexes, with strong evidence for higher ALT values in those with severe vs. low hepatic steatosis starting at 17 years in males and 24 years in females (Figure 2A,B, Table 3). AST levels declined in both sexes until 17 years when they started to increase. In both sexes, strong evidence for differences in AST between those with severe vs. low hepatic steatosis at 24 years were only apparent at 24 years and not in childhood and adolescence (Figure 2C,D, Table 3). In both sexes, GGT values were higher throughout childhood and into adulthood in those with severe vs. low steatosis at 24 years (Figure 2E,F). Adjusting for BMI at 24 years attenuated the estimates of differences in liver enzymes towards the null, although strong differences between those with severe vs. low hepatic steatosis levels remained at 24 years (Table 3). Additionally, adjusting for ethnicity and maternal education did not meaningfully change differences. Differences between all levels of hepatic steatosis and for models 1–3 are presented in Table S1. In males, ALT levels did not differ at any age between those with any vs. no fibrosis at 24 years (Figure 3A). In females, ALT levels were higher at 17 years, which was maintained at 24 years, in those with any vs. no fibrosis at 24 years (Figure 3B).

In the sensitivity analysis, controlling for alcohol intake using the AUDIT-C score, instead of excluding those with hazardous alcohol intake, did not meaningfully change estimates (Table S2).

## 4. Discussion

We found that young adults with severe hepatic steatosis as measured by CAP had a steeper ALT trend from 9 to 24 years compared to those with low hepatic steatosis, with the largest increase occurring from the late teen to young adult years. In males, the differentiation of ALT trends appears to coincide with the beginning of puberty. The association between puberty and increases in steatosis and ALT has been described in previous studies [29,30]. In girls, differences in trend first appeared in late adolescence and with stronger differences occurring in young adulthood. Interestingly, girls with mild/moderate and low steatosis had nearly identical trajectories for ALT, perhaps indicating that ALT is not as sensitive of an indicator in girls, unless they have severe steatosis. NAFLD is known to be a sexually dimorphic disease. Evidence specific to young women indicates that they are better able to partition fatty acids towards ketone body production rather than very-low-density lipoprotein (VLDL)-triacylglycerol packaging compared to young men, leading to protection from dysmetabolic conditions such as NAFLD [31,32].

Concentrations of GGT were consistently higher in those with severe vs. low steatosis over time. However, we found that most associations, particularly for GGT, were attenuated after controlling for BMI at 24 years. The strong association of BMI with ALT and GGT has been previously shown [30,33]. This finding supports the current recommendation that screening for NAFLD be performed using elevated ALT in at-risk populations including overweight and obese children and adolescents [2]. ALT is an inexpensive, widely available, minimally invasive blood test with acceptable sensitivity, making it a useful screening tool in at-risk pediatric populations [2]. Additionally, these results indicate that more research should be conducted to see if GGT should play a larger role for NAFLD screening in childhood and adolescence.

At 24 years, 10.4% of the ALSPAC sample had severe and 11.9% had mild or moderate hepatic steatosis. Abeysekera, et al., using the same cohort, also found a similar 20.7% prevalence of steatosis [34]. This represents a large increase from a NAFLD prevalence of 2.5% (defined as moderate or severe steatosis) in the cohort as measured by ultrasound at the age of 17–18 years [34,35]. This likely partially reflects a true increase in the prevalence. Other possible explanations for the increased prevalence include selection bias and poorer diagnostic ability of the ultrasound technology compared to transient elastography and CAP measures for the assessment of hepatic steatosis. Estimates of the prevalence of NAFLD in adolescents in the US and Australia defined by a variety of methods including autopsy, ultrasound, and ALT, range from 10% to 17% [3,29,36].

Heavy alcohol consumption is of concern in young adults, particularly in the UK [37]. Approximately half of the sample reported hazardous alcohol consumption at 24 years, a known risk factor for hepatic steatosis. To limit potential confounding by alcohol consumption, we excluded all participants with an elevated AUDIT-C score, an instrument used to identify hazardous drinkers. Our criteria led to the exclusion of almost half the sample, therefore, we conducted a sensitivity analysis including participants with any level of drinking and controlling for the AUDIT-C score. We found no meaningful changes to our results. Similar to Abeysekera, et al., we also found no evidence of an association between hazardous alcohol consumption and hepatic steatosis [34]. Future studies should focus on the impact of high alcohol consumption in this population on hepatic steatosis, fibrosis, and other health outcomes.

The largest strength of our study was the use of serial measurements in a large, population-based longitudinal cohort study from childhood to young adulthood. We also used the CAP score based on transient elastography to define hepatic steatosis levels at 24 years, which is a validated and accurate marker of hepatic steatosis in adults [22]. An additional strength is the use of the AUDIT-C score to exclude individuals with hazardous alcohol consumption, increasing our confidence that participants in our analysis with severe hepatic steatosis had non-alcoholic fatty liver disease. The AUDIT-C has been shown to perform well among adolescents with good internal consistency and accuracy [38]. The AUDIT-C has also been shown to be more useful than the AST/ALT ratio, an indicator of alcoholic fatty liver disease (vs. NAFLD), for predicting hazardous drinking [39].

While we did not exclude participants with other liver conditions, a previous report from this population reported that no participants had viral hepatitis or were taking nucleos(t)ide analogues or direct-acting antivirals and very few were taking medications for autoimmune hepatitis [34]. Therefore, we are confident that there was little, if any, confounding by other liver disease.

A limitation of this study was the relatively homogenous nature of the sample, of primarily White ethnicity, and therefore, results may not be generalizable to other populations. We expect that the associations we found would differ in samples with a larger proportion of higher-risk individuals, such as Hispanics [40]. Individuals with adipogenic genes such as Patatin-like phospholipase domain-containing protein 3 (PNPLA3), which is more common in Hispanics, are more susceptible to NAFLD [41]. Additionally, there was differential loss to follow-up within the cohort, whereby females and participants with mothers with higher education were more likely to be followed-up. 

## 5. Conclusions

This is the first description of liver enzyme trends extending from childhood to adulthood and their relation with later hepatic steatosis. ALT trends were associated with hepatic steatosis level in young adulthood. Higher ALT and GGT levels in adolescence were associated with severe hepatic steatosis at 24 years, whereas, prior to puberty, liver enzymes may not be a useful indicator of future risk. The increased testing of liver enzymes in adolescents could strengthen early NAFLD prevention and treatment efforts. There is a need for further quality longitudinal data on the natural history of pediatric NAFLD.

## Figures and Tables

**Table 1:** Demographic and clinical factors at 24 years according to hepatic steatosis level (*n* = 1156) ^1^.

	Total (*n* = 1156)	Low (*n* = 899; 77.8%)	Mild/Moderate (*n* = 137; 11.9%)	Severe (*n* = 120; 10.4%)	*p*-Value
Age	24 (23, 35)	24 (23, 25)	24 (24, 24)	24 (23, 25)	0.751
Sex					
Male	479 (41.4)	348 (38.7)	65 (47.5)	66 (55.0)	
Female	677 (58.6)	551 (61.3)	72 (52.6)	54 (45.0)	<0.001
Ethnic Group					
White	1041 (97.4)	812 (97.8)	124 (96.1)	105 (95.5)	
Other	28 (2.6)	18 (2.2)	5 (3.9)	5 (4.6)	0.086
Mother’s education					
CSE/None	111 (10.4)	83 (10.0)	17 (13.3)	11 (11.5)	
Vocational	70 (6.5)	55 (6.6)	10 (7.8)	5 (4.5)	
O-level	372 (34.8)	280 (33.7)	48 (37.5)	44 (39.6)	
A-level	327 (30.6)	266 (32.0)	32 (25.0)	29 (26.1)	
Degree	190 (17.8)	147 (17.7)	21 (16.4)	22 (19.8)	
BMI, kg/m^2^	23.6 (21.2, 27.0)	22.8 (20.7, 25.3)	27.4 (24.5, 29.9)	32.1 (28.5, 35.9)	0.580
BMI category					
Underweight/Normal	659 (62.4)	608 (73.2)	42 (33.6)	9 (9.0)	<0.001
Overweight	260 (24.6)	178 (21.4)	53 (42.4)	29 (29.0)	
Obese	137 (13.0)	45 (5.4)	30 (24.0)	62 (62.0)	
CAP, dB/m	204 (175, 242)	193 (166, 213)	261 (255, 270)	313 (292.5, 343)	
Fibrosis, kPA	4.6 (3.9, 5.5)	4.6 (3.9, 5.4)	4.5 (3.8, 5.4)	5 (4, 6)	<0.001
Any Fibrosis ^3^	29 (2.6)	21 (2.4)	<5 ^2^	6 (5.3)	
ALT, U/L	20.7 (15.5, 30.0)	19.6 (15.0, 27.9)	20.8 (16.5, 30.8)	35.2 (23.3, 64.0)	<0.001
Elevated ALT	437 (41.0)	308 (36.8)	52 (41.3)	77 (75.5)	0.015
AST, U/L	23.9 (20.5, 29.3)	23.4 (20.3, 28.6)	24.4 (20.0, 28.5)	29.3 (23.9, 36.7)	0.175
GGT, U/L	15.0 (12.0, 21.0)	15.0 (12.0, 19.0)	17.0 (13.0, 22.0)	23.5 (16.0, 35.0)	<0.001
Cholesterol, mmol/L	4.3 (3.8, 4.9)	4.3 (3.8, 4.9)	4.2 (3.9, 4.9)	4.6 (4.1, 5.2)	<0.001
Triglycerides, mmol/L	0.8 (0.6, 1.1)	0.8 (0.6, 1.1)	0.9 (0.7, 1.2)	1.1 (0.8, 1.9)	<0.001
HDL, mmol/L	1.5 (1.2, 1.7)	1.5 (1.3, 1.8)	1.4 (1.1, 1.6)	1.1 (1, 1.4)	<0.001
LDL, mmol/L	2.4 (1.9, 2.9)	2.4 (1.9, 2.9)	2.4 (2.2, 3.2)	2.8 (2.3, 3.2)	0.004
VLDL, mmol/L	0.4 (0.3, 0.5)	0.4 (0.3, 0.5)	0.4 (0.3, 0.5)	0.5 (0.4, 0.8)	<0.001
Insulin, mu/L	7.7 (5.4, 11.3)	7 (5, 9.7)	10.8 (7.2, 15.2)	16.6 (10.8, 25.1)	<0.001
Glucose, mmol/L	5.3 (5.0, 5.6)	5.3 (5, 5.6)	5.4 (5.1, 5.7)	5.5 (5.3, 5.8)	<0.001

^1^ Values represent the median (IQR) or number of participants (%). Chi-squared tests were used to compare the differences between categorical variables. For cell counts <5, Fisher’s exact tests were used. Kruskal–Wallis tests were used to compare the differences across steatosis levels for continuous variables. Some variables had missing values: ethnic group (*n* = 87), mother’s highest education level (*n* = 86), BMI (*n* = 9), fibrosis (*n* = 56), and biomarker values (*n* = 91). ^2^ Groups with less than five participants are expressed as *n* < 5 in line with the Avon Longitudinal Study of Parents and Children (ALSPAC) confidentiality policy. ^3^ Any fibrosis includes those with portal fibrosis, septa, or cirrhosis (F2–F4, >7.9 kPA). Abbreviations: CSE = certificate of secondary education, BMI = body mass index, CAP = controlled attenuation parameter, ALT = alanine aminotransferase, AST = aspartate aminotransferase, GGT = gamma-glutamyl transferase, HDL = high-density lipoprotein, LDL = low-density lipoprotein, VLDL = very low-density lipoprotein.

**Table 2:** ALT, AST, and GGT by age and hepatic steatosis at 24 years (*n* = 479 males, 677 females) ^1^.

	9 Years	15 Years	17 Years	24 Years	*p*-Value
**Hepatic Steatosis**	**ALT, U/L**				
Males					<0.001
Low	11.8 (9.0, 15.3)	16.9 (13.5, 20.6)	15.0 (12.3, 20.6)	24.4 (18.2, 32.8)	
Mild/Moderate	11.8 (8.5, 16.0)	17.5 (14.1, 22.7)	17.7 (13.0, 21.2)	28.2 (20.6, 39.7)	
Severe	11.9 (8.8, 14.0)	17.9 (14.2, 23.5)	22.6 (15.3, 30.4)	46.8 (34.6, 73.3)	
Females					<0.001
Low	11.7 (9.2, 14.8)	13.5 (10.6, 17.0)	13.7 (11.1, 17.2)	17.2 (13.4, 23.0)	
Mild/Moderate	11.2 (8.8, 13.9)	13.8 (10.9, 16.1)	14.5 (11.3, 20.1)	16.9 (14.7, 23.6)	
Severe	12.0 (8.6, 15.0)	13.1 (9.9, 19.2)	15.9 (12.3, 27.2)	25.4 (17.9, 32.9)	
	**AST, U/L**				
Males					<0.001
Low	32.1 (28.4, 35.6)	22.9 (19.6, 26.9)	20.2 (17.2, 23.4)	26.1 (21.7, 31.2)	
Mild/Moderate	30.8 (28.0, 34.2)	23.1 (20.8, 29.4)	20.5 (17.5, 27.1)	26.5 (24.1, 31.6)	
Severe	32.0 (28.2, 35.3)	22.9 (19.9, 27.4)	21.7 (18.2, 25.6)	33.8 (25.9, 38.7)	
Females					<0.001
Low	30.5 (27.1, 34.7)	19.5 (17.2, 22.0)	18.6 (16.2, 22.0)	22.2 (19.5, 26.0)	
Mild/Moderate	29.4 (27.1, 33.3)	18.5 (16.4, 21.7)	17.8 (15.5, 22.2)	21.9 (18.9, 26.0)	
Severe	29.1 (25.8, 32.2)	19.2 (15.7, 22.3)	17.6 (16.6, 21.9)	25.5 (22.2, 31.3)	
	**GGT, U/L**				
Males					<0.001
Low	15.0 (14.0, 18.0)	16.0 (14.0, 18.0)	17.0 (14.0, 21.0)	16.0 (14.0, 21.0)	
Mild/Moderate	17.0 (14.0, 22.0)	17.0 (15.0, 23.0)	21.0 (16.0, 25.0)	20.5 (15.0, 30.5)	
Severe	17.0 (14.0, 22.0)	19.0 (16.0, 23.0)	21.0 (16.0, 32.0)	27.0 (19.0, 38.0)	
Females					0.002
Low	15.0 (13.0, 18.0)	14.0 (11.0, 16.0)	14.0 (12.0, 18.0)	14.0 (11.0, 18.0)	
Mild/Moderate	16.0 (14.0, 20.0)	14.0 (12.0, 19.0)	17.0 (13.0, 22.0)	14.0 (12.0, 18.0)	
Severe	18.0 (14.0, 22.0)	14.0 (12.0, 19.0)	17.0 (13.0, 20.0)	19.0 (14.0, 31.0)	

^1^ Median (IQR). *p*-value from a type 3 test of fixed effects interaction between age and hepatic steatosis level in model 1. Steatosis is defined from the controlled attenuation parameter scores: low (<248 dB/m), mild/moderate (248–279 dB/m), severe (>279 dB/m). Abbreviations: ALT = alanine aminotransferase, AST = aspartate aminotransferase, GGT = gamma-glutamyl transferase.

**Table 3:** Geometric mean ratios and 95% CIs of liver enzymes for low vs. severe hepatic steatosis level at each age (years) and by sex ^1^.

	Males (*n* = 479)		Females (*n* = 677)	
**ALT**	**Unadjusted**	**Fully Adjusted ^2^**	**Unadjusted**	**Fully Adjusted ^2^**
9 years	1.02 (0.82, 1.27)	1.15 (0.90, 1.48)	1.02 (0.82, 1.26)	1.13 (0.89, 1.44)
15 years	0.89 (0.74, 1.07)	1.01 (0.81, 1.26)	1.01 (0.83, 1.24)	1.15 (0.91, 1.45)
17 years	0.69 (0.57, 0.85)	0.8 (0.64, 1.01)	0.81 (0.65, 1.01)	1.01 (0.79, 1.29)
24 years	0.53 (0.42, 0.66)	0.63 (0.5, 0.81)	0.67 (0.54, 0.84)	0.75 (0.59, 0.96)
**AST**				
9 years	1.01 (0.91, 1.13)	1.03 (0.91, 1.18)	1.08 (0.95, 1.22)	1.08 (0.93, 1.24)
15 years	1.01 (0.89, 1.15)	1.02 (0.88, 1.19)	1.07 (0.94, 1.22)	1.02 (0.88, 1.18)
17 years	0.92 (0.79, 1.06)	0.93 (0.79, 1.10)	0.99 (0.87, 1.13)	1.02 (0.88, 1.18)
24 years	0.81 (0.70, 0.94)	0.86 (0.72, 1.01)	0.86 (0.75, 0.97)	0.84 (0.73, 0.97)
**GGT**				
9 years	0.87 (0.76, 1.00)	1.01 (0.86, 1.18)	0.86 (0.75, 0.99)	1.00 (0.85, 1.18)
15 years	0.82 (0.71, 0.96)	0.99 (0.85, 1.16)	0.87 (0.75, 1.02)	0.99 (0.82, 1.19)
17 years	0.76 (0.66, 0.89)	0.90 (0.75, 1.07)	0.87 (0.71, 1.07)	1.03 (0.82, 1.30)
24 years	0.62 (0.51, 0.76)	0.73 (0.59, 0.89)	0.66 (0.53, 0.83)	0.77 (0.61, 0.98)

^1^ Steatosis is defined from controlled attenuation parameter scores: low (<248 dB/m), mild/moderate (248–279 dB/m), severe (>279 dB/m). ^2^ Adjusted for BMI at 24 years, maternal ethnicity and education. Abbreviations: ALT = alanine aminotransferase, AST = aspartate aminotransferase, GGT = gamma-glutamyl transferase.

## Supplementary Materials

The following are available online at https://www.mdpi.com/2227-9067/7/9/117/s1, Table S1: Geometric mean ratios and 95% CIs of liver enzymes for hepatic steatosis levels at each age stratified by sex, Table S2: Geometric mean ratios and 95% CIs of liver enzymes for hepatic steatosis levels at each age stratified by sex including participants regardless of AUDIT-C.
