# Supplementary material for: ALT Trends through Childhood and Adolescence Associated with Hepatic Steatosis at 24 Years: A Population-Based UK Cohort Study
Source: Children (Basel). 2020 Sep 1;7(9):117. doi: 10.3390/children7090117 (PMC7552746; doi:10.3390/children7090117)
Supplement: Supplementary file 1 [file children-07-00117-s001.zip › children-906510-supplementary.docx]

**Supplement**

| **Table S1. Geometric mean ratios and 95% CIs of liver enzymes for hepatic steatosis levels at each age stratified by sex** | | | | | | | |
| --- | --- | --- | --- | --- | --- | --- | --- |
|  |  | **Males (n=479)** | | | **Females (n=677)** | | |
| **Age** | **Steatosis** | **Model 1** | **Model 2** | **Model 3** | **Model 1** | **Model 2** | **Model 3** |
| **ALT** | | | | | | | |
| 9 | Low v Med | 0.95 (0.77, 1.18) | 1.05 (0.84, 1.31) | 1.02 (0.81, 1.27) | 1.06 (0.87, 1.29) | 1.10 (0.90, 1.34) | 1.10 (0.89, 1.36) |
| 9 | Low v Severe | 1.02 (0.82, 1.27) | 1.23 (0.96, 1.56) | 1.15 (0.9, 1.48) | 1.02 (0.82, 1.26) | 1.13 (0.90, 1.43) | 1.13 (0.89, 1.44) |
| 9 | Med v Severe | 1.07 (0.81, 1.42) | 1.17 (0.87, 1.56) | 1.13 (0.85, 1.52) | 0.96 (0.73, 1.27) | 1.03 (0.78, 1.37) | 1.03 (0.76, 1.38) |
| 15 | Low v Med | 1.00 (0.82, 1.23) | 1.10 (0.89, 1.35) | 1.12 (0.91, 1.38) | 1.02 (0.84, 1.23) | 1.07 (0.88, 1.30) | 1.09 (0.88, 1.34) |
| 15 | Low v Severe | 0.89 (0.74, 1.07) | 1.06 (0.86, 1.31) | 1.01 (0.81, 1.26) | 1.01 (0.83, 1.24) | 1.15 (0.92, 1.43) | 1.15 (0.91, 1.45) |
| 15 | Med v Severe | 0.88 (0.69, 1.14) | 0.97 (0.75, 1.26) | 0.90 (0.69, 1.18) | 1.00 (0.77, 1.30) | 1.07 (0.82, 1.41) | 1.06 (0.80, 1.41) |
| 17 | Low v Med | 0.89 (0.71, 1.11) | 0.97 (0.78, 1.21) | 0.97 (0.78, 1.21) | 0.93 (0.76, 1.14) | 0.98 (0.80, 1.20) | 1.00 (0.81, 1.23) |
| 17 | Low v Severe | **0.69 (0.57, 0.85)** | 0.84 (0.67, 1.04) | 0.8 (0.64, 1.01) | 0.81 (0.65, 1.01) | 0.92 (0.72, 1.17) | 1.01 (0.79, 1.29) |
| 17 | Med v Severe | 0.78 (0.59, 1.03) | 0.86 (0.66, 1.13) | 0.83 (0.63, 1.09) | 0.87 (0.66, 1.16) | 0.94 (0.70, 1.25) | 1.01 (0.75, 1.36) |
| 24 | Low v Med | 0.84 (0.68, 1.05) | 0.92 (0.74, 1.14) | 0.92 (0.74, 1.14) | 0.98 (0.81, 1.19) | 1.03 (0.85, 1.24) | 1.04 (0.86, 1.27) |
| 24 | Low v Severe | **0.53 (0.42, 0.66)** | **0.63 (0.50, 0.80)** | **0.63 (0.50, 0.81)** | **0.67 (0.54, 0.84)** | **0.74 (0.58, 0.95)** | **0.75 (0.59, 0.96)** |
| 24 | Med v Severe | **0.63 (0.47, 0.83)** | **0.69 (0.52, 0.91)** | **0.69 (0.52, 0.92)** | **0.69 (0.52, 0.91)** | **0.73 (0.55, 0.97)** | **0.72 (0.54, 0.96)** |
| **AST** |  | | | | | | |
| 9 | Low v Med | 0.99 (0.89, 1.11) | 1.01 (0.90, 1.14) | 1 (0.89, 1.13) | 1.03 (0.92, 1.16) | 1.03 (0.91, 1.16) | 1.03 (0.91, 1.16) |
| 9 | Low v Severe | 1.01 (0.91, 1.13) | 1.05 (0.92, 1.19) | 1.03 (0.91, 1.18) | 1.08 (0.95, 1.22) | 1.08 (0.94, 1.24) | 1.08 (0.93, 1.24) |
| 9 | Med v Severe | 1.02 (0.88, 1.18) | 1.04 (0.89, 1.20) | 1.03 (0.88, 1.20) | 1.04 (0.89, 1.22) | 1.05 (0.88, 1.24) | 1.05 (0.88, 1.25) |
| 15 | Low v Med | 0.98 (0.85, 1.13) | 1.00 (0.86, 1.15) | 1.00 (0.86, 1.16) | 1.04 (0.92, 1.18) | 1.04 (0.91, 1.18) | 1.05 (0.92, 1.20) |
| 15 | Low v Severe | 1.01 (0.89, 1.15) | 1.05 (0.91, 1.21) | 1.02 (0.88, 1.19) | 1.07 (0.94, 1.22) | 1.06 (0.92, 1.22) | 1.02 (0.88, 1.18) |
| 15 | Med v Severe | 1.03 (0.86, 1.23) | 1.05 (0.88, 1.26) | 1.02 (0.85, 1.24) | 1.03 (0.87, 1.22) | 1.02 (0.86, 1.22) | 0.97 (0.81, 1.16) |
| 17 | Low v Med | 0.97 (0.83, 1.14) | 0.99 (0.84, 1.16) | 0.99 (0.84, 1.17) | 1.03 (0.91, 1.16) | 1.02 (0.9, 1.16) | 1.04 (0.92, 1.18) |
| 17 | Low v Severe | 0.92 (0.79, 1.06) | 0.95 (0.81, 1.11) | 0.93 (0.79, 1.10) | 0.99 (0.87, 1.13) | 0.99 (0.85, 1.14) | 1.02 (0.88, 1.18) |
| 17 | Med v Severe | 0.94 (0.77, 1.15) | 0.96 (0.79, 1.18) | 0.94 (0.76, 1.16) | 0.96 (0.81, 1.14) | 0.97 (0.81, 1.15) | 0.98 (0.83, 1.17) |
| 24 | Low v Med | 0.96 (0.83, 1.11) | 0.97 (0.84, 1.13) | 0.97 (0.84, 1.12) | 1.03 (0.92, 1.15) | 1.02 (0.91, 1.15) | 1.03 (0.92, 1.16) |
| 24 | Low v Severe | **0.81 (0.70, 0.94)** | **0.84 (0.71, 0.98)** | 0.86 (0.72, 1.01) | **0.86 (0.75, 0.97)** | **0.84 (0.73, 0.97)** | **0.84 (0.73, 0.97)** |
| 24 | Med v Severe | 0.85 (0.70, 1.02) | 0.86 (0.71, 1.04) | 0.88 (0.73, 1.08) | **0.83 (0.71, 0.98)** | **0.82 (0.69, 0.97)** | **0.81 (0.69, 0.97)** |
| **GGT** |  | | | | | | |
| 9 | Low v Med | 0.95 (0.83, 1.09) | 1.03 (0.90, 1.19) | 1.02 (0.89, 1.18) | 0.94 (0.83, 1.07) | 0.99 (0.87, 1.12) | 0.99 (0.87, 1.13) |
| 9 | Low v Severe | **0.87 (0.76, 1.00)** | 1.01 (0.86, 1.18) | 1.01 (0.86, 1.18) | 0.86 (0.75, 0.99) | 0.97 (0.82, 1.13) | 1.00 (0.85, 1.18) |
| 9 | Med v Severe | 0.91 (0.76, 1.09) | 0.97 (0.81, 1.17) | 0.98 (0.82, 1.18) | 0.91 (0.76, 1.09) | 0.98 (0.81, 1.17) | 1.01 (0.84, 1.22) |
| 15 | Low v Med | 0.89 (0.75, 1.04) | 0.95 (0.81, 1.12) | 0.97 (0.84, 1.13) | 0.95 (0.82, 1.09) | 1 (0.86, 1.16) | 1.01 (0.86, 1.19) |
| 15 | Low v Severe | **0.82 (0.71, 0.96)** | 0.95 (0.81, 1.13) | 0.99 (0.85, 1.16) | 0.87 (0.75, 1.02) | 0.98 (0.82, 1.17) | 0.99 (0.82, 1.19) |
| 15 | Med v Severe | 0.93 (0.76, 1.14) | 1.00 (0.82, 1.23) | 1.02 (0.85, 1.23) | 0.92 (0.75, 1.13) | 0.98 (0.8, 1.21) | 0.98 (0.78, 1.22) |
| 17 | Low v Med | **0.84 (0.71, 0.99)** | 0.90 (0.77, 1.06) | 0.91 (0.77, 1.07) | 0.93 (0.78, 1.12) | 0.98 (0.82, 1.18) | 1.03 (0.85, 1.26) |
| 17 | Low v Severe | **0.76 (0.66, 0.89)** | 0.89 (0.75, 1.05) | 0.90 (0.75, 1.07) | 0.87 (0.71, 1.07) | 0.99 (0.8, 1.23) | 1.03 (0.82, 1.30) |
| 17 | Med v Severe | 0.91 (0.74, 1.12) | 0.98 (0.80, 1.20) | 0.99 (0.80, 1.22) | 0.93 (0.72, 1.21) | 1.01 (0.77, 1.31) | 1.00 (0.75, 1.32) |
| 24 | Low v Med | **0.80 (0.66, 0.97)** | 0.86 (0.72, 1.04) | 0.87 (0.72, 1.04) | 0.97 (0.80, 1.16) | 1.02 (0.84, 1.23) | 1.05 (0.86, 1.28) |
| 24 | Low v Severe | **0.62 (0.51, 0.76)** | **0.72 (0.59, 0.88)** | **0.73 (0.59, 0.89)** | **0.66 (0.53, 0.83)** | **0.76 (0.60, 0.95)** | **0.77 (0.61, 0.98)** |
| 24 | Med v Severe | **0.78 (0.60, 1.00)** | 0.84 (0.66, 1.07) | 0.84 (0.66, 1.07) | **0.69 (0.52, 0.90)** | **0.74 (0.56, 0.98)** | **0.74 (0.55, 0.98)** |
| Note: Medium indicates mild or moderates steatosis. Steatosis is defined from controlled attenuation parameter scores: low (<248 dB/m), mild/moderate (248-279 dB/m), severe (>279 dB/m). Model 1 is unadjusted, model 2 is adjusted for BMI at 24 years, and model 3 is additionally adjusted for maternal ethnicity and education. Ratios that do not cross 1.0 are bolded. | | | | | | | |

| **Table S2. Geometric mean ratios and 95% CIs of liver enzymes for hepatic steatosis levels at each age stratified by sex including participants regardless of AUDIT-C score.** | | | | | | | |
| --- | --- | --- | --- | --- | --- | --- | --- |
|  |  | **Males (n=1038)** | | | **Females (n=1513)** | | |
| **Age** | **Steatosis** | **GMR** | **95% CI** | | **GMR** | **95% CI** | |
| **ALT** | | | | | | | |
| 9 | Low v Med | 1.00 | 0.86 | 1.17 | 1.03 | 0.90 | 1.18 |
| 9 | Low v Severe | 0.93 | 0.79 | 1.09 | 0.92 | 0.79 | 1.06 |
| 9 | Med v Severe | 0.93 | 0.75 | 1.14 | 0.89 | 0.74 | 1.08 |
| 15 | Low v Med | 0.99 | 0.85 | 1.16 | 0.99 | 0.87 | 1.12 |
| 15 | Low v Severe | 0.86 | 0.74 | 0.99 | 0.97 | 0.84 | 1.11 |
| 15 | Med v Severe | 0.87 | 0.71 | 1.05 | 0.98 | 0.82 | 1.17 |
| 17 | Low v Med | 0.93 | 0.80 | 1.09 | 0.97 | 0.84 | 1.11 |
| 17 | Low v Severe | 0.77 | 0.66 | 0.89 | 0.89 | 0.76 | 1.03 |
| 17 | Med v Severe | 0.82 | 0.67 | 1.00 | 0.92 | 0.75 | 1.12 |
| 24 | Low v Med | 0.90 | 0.78 | 1.04 | 0.96 | 0.83 | 1.10 |
| 24 | Low v Severe | 0.60 | 0.52 | 0.70 | 0.70 | 0.60 | 0.82 |
| 24 | Med v Severe | 0.67 | 0.55 | 0.81 | 0.73 | 0.60 | 0.89 |
| **AST** |  | | | | | | |
| 9 | Low v Med | 1.01 | 0.93 | 1.09 | 1.04 | 0.96 | 1.12 |
| 9 | Low v Severe | 1.00 | 0.91 | 1.09 | 1.03 | 0.95 | 1.12 |
| 9 | Med v Severe | 0.99 | 0.89 | 1.11 | 0.99 | 0.89 | 1.10 |
| 15 | Low v Med | 0.99 | 0.89 | 1.10 | 1.02 | 0.94 | 1.11 |
| 15 | Low v Severe | 0.98 | 0.88 | 1.08 | 1.01 | 0.92 | 1.11 |
| 15 | Med v Severe | 0.99 | 0.86 | 1.13 | 0.99 | 0.88 | 1.11 |
| 17 | Low v Med | 1.00 | 0.90 | 1.11 | 1.04 | 0.95 | 1.14 |
| 17 | Low v Severe | 0.96 | 0.87 | 1.06 | 1.01 | 0.92 | 1.11 |
| 17 | Med v Severe | 0.96 | 0.84 | 1.10 | 0.97 | 0.86 | 1.09 |
| 24 | Low v Med | 1.02 | 0.92 | 1.13 | 1.05 | 0.96 | 1.16 |
| 24 | Low v Severe | 0.86 | 0.77 | 0.96 | 0.89 | 0.80 | 0.99 |
| 24 | Med v Severe | 0.84 | 0.73 | 0.97 | 0.85 | 0.74 | 0.97 |
| **GGT** |  | | | | | | |
| 9 | Low v Med | 0.97 | 0.88 | 1.06 | 0.94 | 0.86 | 1.03 |
| 9 | Low v Severe | 0.90 | 0.81 | 0.99 | 0.89 | 0.80 | 0.98 |
| 9 | Med v Severe | 0.93 | 0.82 | 1.05 | 0.94 | 0.83 | 1.07 |
| 15 | Low v Med | 0.94 | 0.84 | 1.04 | 1.01 | 0.91 | 1.11 |
| 15 | Low v Severe | 0.82 | 0.74 | 0.91 | 0.85 | 0.77 | 0.95 |
| 15 | Med v Severe | 0.88 | 0.76 | 1.01 | 0.85 | 0.74 | 0.97 |
| 17 | Low v Med | 0.90 | 0.80 | 1.02 | 0.94 | 0.83 | 1.06 |
| 17 | Low v Severe | 0.77 | 0.68 | 0.86 | 0.88 | 0.77 | 1.00 |
| 17 | Med v Severe | 0.85 | 0.73 | 0.99 | 0.94 | 0.79 | 1.11 |
| 24 | Low v Med | 0.82 | 0.72 | 0.95 | 0.91 | 0.79 | 1.04 |
| 24 | Low v Severe | 0.65 | 0.57 | 0.75 | 0.72 | 0.62 | 0.84 |
| 24 | Med v Severe | 0.79 | 0.66 | 0.95 | 0.80 | 0.66 | 0.96 |
| Note: Medium indicates mild or moderates steatosis. Steatosis is defined from controlled attenuation parameter scores: low (<248 dB/m), mild/moderate (248-279 dB/m), severe (>279 dB/m). Model controls for AUDIT-C score. | | | | | | | |
